# Processing Method and Performance Evaluation of Flame-Retardant Corrugated Sandwich Panel

**DOI:** 10.3390/polym16050696

**Published:** 2024-03-04

**Authors:** Yiliang Sun, Jingwen Li, Boming Zhang

**Affiliations:** 1 School of Materials Science and Engineering, Beihang University, Beijing 100191, China; ylsun@buaa.edu.cn (Y.S.);; 2 Institute of Advanced Materials, Shandong Institutes of Industrial Technology, Jinan 250102, China

**Keywords:** flame retardant, corrugated sandwich panel, thermoplastic composites, continuous fiber

## Abstract

In this study, in order to expand the engineering application range of thermoplastic corrugated sheets, flame-retardant thermoplastic corrugated sheets were prepared by the thermoplastic molding method. Based on our previous research results, we prepared flame-retardant prepreg tapes with the flame retardant addition accounting for 15%, 20%, and 25% of the resin matrix. Then, we prepared flame-retardant thermoplastic corrugated sandwich panels with corresponding flame retardant addition amounts. The limiting oxygen index test, vertical combustion test, cone calorimetry test, and mechanical property test were carried out on each group of samples and control group samples. The results showed that when the flame retardant was added at 25%, the flame retardant level could reach the V0 level. Compared with the control group, the flexural strength and flexural modulus decreased by 2.6%, 14.1%, and 19.9% and 7.3%, 16.1%, and 21.9%, respectively. When the amount of flame retardant was 15%, 20%, and 25%, respectively, the total heat release decreased by 16.3%, 23.5%, and 34.1%, and the maximum heat release rate decreased by 12.5%, 32.4%, and 37.4%, respectively.

## 1. Introduction

Compared with traditional thermosetting composites [1,2,3], continuous-fiber-reinforced thermoplastic composites are recyclable, in line with the concepts of low carbon and environmental protection, and also afford a fast molding speed and good impact toughness. Continuous-fiber-reinforced thermoplastic prepregs have no storage conditions. Therefore, the research and applications of thermoplastic composites have attracted increasing attention in recent years [4].

In the thermoplastic resin matrix, polypropylene has the advantages of a low molding temperature, low price, good chemical stability, and excellent comprehensive performance [5]. The application fields and market for continuous fiber-reinforced polypropylene composites are also gradually expanding. The flammable characteristics of polypropylene have led to its limitations in many application scenarios; thus, modifying polypropylene with flame retardants has important practical significance for expanding the application range of polypropylene and its composites. Research on flame retardants for polypropylene composites is relatively comprehensive and mature. However, according to a literature review, research on continuous fiber reinforcement needs to be further expanded and deepened. The flame retardancy of continuous fiber-reinforced polypropylene shares some similarities with that of the polypropylene matrix, and there are also many differences that have not received special attention in previous research [6]. We conducted extensive research on the flame retardancy of continuous glass fiber (CGF)-reinforced polypropylene. Building on previous research [7], we further investigated the structural applications of continuous-fiber-reinforced flame-retardant polypropylene. The corrugated sandwich panel is a representative structure [8,9,10,11,12]. Corrugated structures are simple to prepare, have strong bearing capacities, and are widely used in aerospace, marine vessels, packaging, and other fields [13,14,15,16]. Compared with other structural forms, such as the honeycomb sandwich structure, the corrugated structure is characterized by the fact that the available overall space of the corrugated structure is larger than that of the honeycomb structure. This feature can facilitate the subsequent use of corrugated sandwich panels, such as filling the corrugated space with thermal insulation to increase thermal insulation performance and also using its space to place other components. It can be said that the corrugated sandwich structure can provide more space that can be used, which is also a feature and advantage compared with other sandwich core structures. The feature can provide designers and engineers with more design possibilities. For example, when the corrugated sandwich panel structure is applied to the new energy vehicle industry, the battery part can be put into the corrugated space to realize the integration of structure and function.

At present, based on a literature survey, there is no relevant research on continuous fiber-reinforced polypropylene flame-retardant corrugated sandwich panels [17,18]. However, few studies have paid attention to the flame retardancy of continuous fiber thermoplastic corrugated sandwich panels, and whether the flame retardant performance can meet the corresponding requirements is the key to whether it can meet the needs of use scenarios. Therefore, this study focuses on further exploring the preparation and performance of a flame-retardant corrugated plate on the basis of previous research on continuous fiber-reinforced polypropylene flame-retardant prepregs.

In this study, we prepare a continuous fiber-reinforced flame-retardant polypropylene prepreg tape and further prepare a flame-retardant prepreg tape on flame-retardant corrugated panels. The process possibility of preparing corrugated core materials by thermoplastic molding was verified. The effect of the addition of flame retardant on the mechanical properties of composite materials was compared by the bending mechanical properties test. The flame retardant performance of corrugated panels is evaluated. The amount of flame retardant added to meet the different flame-retardant performance levels was determined.

## 2. Materials and Methods

### 2.1. Raw Materials and Test Equipment

Polypropylene (Bx3900), plastic granules with a melt flow index of 100.0 g/10 min (230 °C, 2.16 kg), was obtained from SK (Corporation: Seoul, Republic of Korea). Polypropylene (MF650X), plastic granules with a melt flow index of 1200.0 g/10 min (230 °C, 2.16 kg), was obtained from LyondellBasell (Corporation: Rotterdam, The Netherlands). Maleic anhydride-grafted polypropylene (MAPP), MF650X, plastic granules with a melt flow index of 20.0 g/10 min (230 °C, 2.16 kg), was obtained from Exxon Mobil Co., Ltd. (Brussels, Belgium). The intumescent flame retardant(IFR) was purchased from Xinxiu Chemical Co., Ltd. (Xinxiang, China). The Continuous fiberglass yarn (4305S), pre-treated by sizing agent and dedicated to the Polypropylene resin, was obtained from Chongqing Polycomp International Corporation (Chongqing, China).

In order to make the material and equipment information used in the experiment easily accessible and readable, it has been presented in the form of a table. Information on the materials used in the experiment is listed in Table 1. The information on test equipment is listed in Table 2.

### 2.2. Preparation of Flame-Retardant Prepreg Tape

We first prepared flame-retardant-modified polypropylene by melt-blending and then used a self-designed and assembled prepreg tape production line to prepare the flame-retardant prepreg tape with the fiber, as described in our previous work. In the preparation of the continuous glass fiber-reinforced flame-retardant prepreg tape, the key process parameters in the prepreg belt process were adjusted to obtain a flame-retardant prepreg belt with excellent performance for the subsequent related experiments.

### 2.3. Preparation of Flame-Retardant Corrugated Sandwich Panels

After preparation, the flame-retardant prepreg was cut to a certain size and used to form the corrugated plate panel and core material. The preparation of the flame-retardant corrugated plate is detailed below.

#### 2.3.1. Preparation of Upper and Lower Panels of Corrugated Plates by Molding Method

The skin of the corrugated plate was prepared by the molding method. The prepared flame-retardant prepreg was cut and spliced into a rectangular shape with dimensions of 320 mm × 120 mm and laid in the middle of two layers of aluminum plate according to the designed laying order, then heated on the heating plate for 5 min. The resin in the prepreg in the heating plate was in a completely molten state, and the aluminum plate was immediately placed in a molding machine for molding. The molding pressure was set to 5 MPa, the temperature of the molding machine was set to 80 °C, and the heat preservation and pressure holding time was 5 min.

#### 2.3.2. Preparation of Core Material for Corrugated Plate by Roller Pressing Method

**Figure 1:**
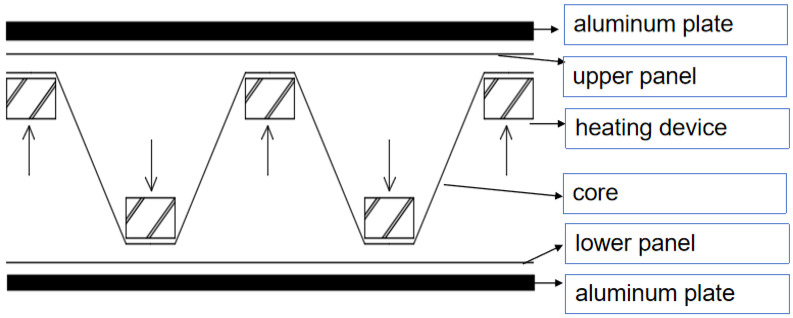
Bonding between the corrugated core material and the panels.

The corrugated core material was prepared using a roller-pressing method. The flame-retardant prepreg tape was wound around the unwinding reel. The unwinding device is composed of an unwinding shaft, drive motor, and other parts. The motor drives the unwinding shaft and rotates to realize the continuous motion of the prepreg belt. The prepreg belt was heated by the heating device. The temperature of the heating device was set to 250 °C. The resin in the prepreg belt in a molten state was passed through the roll mold, and the corrugated plate core material structure was formed. The overall molding process is shown in Figure 1.

#### 2.3.3. Hot Melt Method of Bonding Upper and Lower Panels and Corrugated Core Materials

Based on the structural characteristics of the corrugated core materials, combined with the advantages of the thermoplastic composite materials for secondary processing, a bonding device for connecting the corrugated core materials with the upper and lower panels was designed. The bonding device is shown in Figure 1, where the prepared corrugated core material was placed in the middle of the upper and lower panels. The outside of the material (the upper and lower aluminum plates) can be heated, and the heat-shaping rod is placed inside the core material. The heating temperature of the heating rod was set to 220 °C, and the heating temperature of the upper and lower heating aluminum plates was set to 220 °C. After the core material and the heating position of the upper and lower panels were molten, pressure was applied through the heating rod to complete the bonding molding of the corrugated core material and the panel.

**Figure 2:**
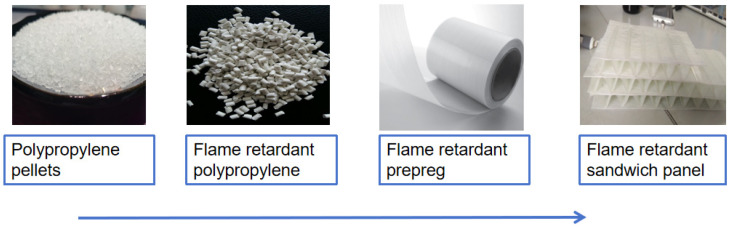
Schematic diagram of the experimental process.

Figure 2 visually shows the overall preparation process from polypropylene pellets to flame-retardant corrugated sandwich panels.

#### 2.3.4. Water Cutting Method for Cutting Specimens

After preparing the flame-retardant corrugated plate, the specimen was cut by water cutting to avoid melting at the edge of the cutting material caused by the heat generated during the process of cutting the thermoplastic composites.

### 2.4. Performance Test

#### 2.4.1. Limiting Oxygen Index Test

The limiting oxygen index (LOI) method, also known as the LOI or critical oxygen index method, was proposed by Fenimore and Martin in 1966 to evaluate the combustion performance of plastics and textile materials. The oxygen index test is a good indicator of the combustion performance of a material. The combustion performance can be quantitatively evaluated with numerical results to a certain extent. The advantages are: the test is simple and the experimental cost is relatively low. This method has been widely used in evaluating the combustion characteristics and fire performance of materials.

The limiting oxygen index is defined as the minimum oxygen concentration at which the tested sample can maintain combustion under specified experimental conditions, that is, the lowest volume percentage of oxygen in the combustion environment of a gas-oxygen-nitrogen mixture during the experiment, expressed as follows:

$$\mathrm{LOI}=\frac{\left[O_{2}\right]}{\left[O_{2}]+[N_{2}\right]}\times 100\%,$$

where [O_2_] and [N_2_] are the volumetric flow rates of oxygen and nitrogen, respectively.

#### 2.4.2. Vertical Combustion Test

Plastic combustion grade tests are divided into horizontal and vertical combustion tests and are commonly used to evaluate the combustion performance of materials. The UL94 vertical combustion test method divides the difficulty of material combustion into V-0, V-1, and V-2 grades. The specific test methods and grade judgment standards were referenced from the test standard ASTM D3801-20a protocol (Standard Test Method for Measuring the Comparative Burning Characteristics of Solid Plastics in a Vertical Position). Vertical burning tests were conducted using a vertical burning test instrument (YK-Y0142) (Yaoke, Nanjing, China) with dimensions of 130 mm× 13 mm × 3.0 mm.

#### 2.4.3. Cone Calorimetry Test

A cone calorimeter is used to determine the amount of heat released during combustion by measuring the oxygen consumed by the material during combustion, which in turn determines the rate of heat release of the material during the combustion test. Cone calorimetry tests (CCTs) were conducted using a cone calorimeter (Fire Testing Technology, Leeds, UK) with a heat flux of 50 kW/m^2^ according to the ISO 5660 standard. Each specimen measured 100 mm × 100 mm × 12 mm.

#### 2.4.4. Mechanical Properties Test

The mechanical property tests were performed on Changchun Kexin WDW-100 Universal Mechanical Testing Machine. According to ASTM D7264 standard, three-point bending property tests were carried out on each specimen at a beam moving speed of 2 mm/min to obtain the bending strength and bending modulus of the composites.

## 3. Results and Discussion

### 3.1. Limiting Oxygen Index and Combustion Rate Test

**Figure 3:**
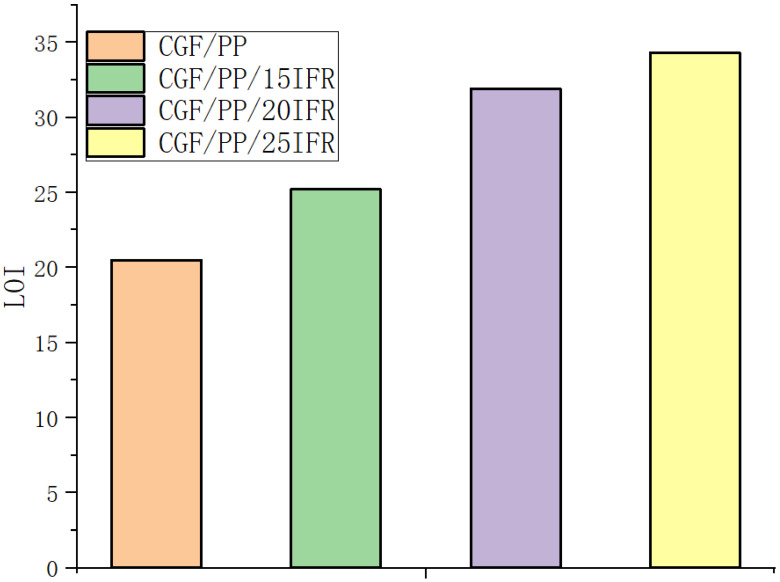
Comparison of limiting oxygen indices of each group of specimens.

The results of the limiting oxygen index test are shown in Figure 3. The limiting oxygen index of the blank control group without flame retardant addition is 20.5. When 15, 20, and 25% flame retardant were each added, the limiting oxygen index was 25.2, 31.9, and 34.3, corresponding to an increase of 22.9, 55.6, and 67.3%, respectively. The test results indicate that as the amount of added flame retardant increased, the limiting oxygen index increased. The limiting oxygen index was largest when the content of the flame retardant was increased from 15% to 20%, moving from 22.9% to 55.6%; after the added amount exceeded 20%, the limiting oxygen index increased to a lesser extent. When the amount of added flame retardant was increased from 20% to 25%, the limiting oxygen index increased from 55.6% to 67.3%. This phenomenon can be explained by considering the combustion mechanism. With a flame-retardant content of 15%, the expanded carbon layer formed after the combustion of the material is relatively fluffy and not dense; thus, the flame-retardant effect is not good. With the addition of 20% flame retardant, the expanded carbon layer formed by the sample during combustion is more compact, the flame-retardant effect is further improved, and the data show that the limiting oxygen index is greatly improved. When the flame-retardant content is further increased, the density of the expanded carbon layer formed after the combustion of the sample is limited. Thus, the limiting oxygen index increased to a lesser extent.

The results of the combustion grade test are shown in Table 3. When no flame retardant was added, because of the continuous fiber used as the reinforcement, the glass fiber did not burn during the combustion process, and the specimen did not exhibit droplet behavior during combustion, which is different from the combustion of the polypropylene matrix spline. With an increase in the amount of flame retardant to 25%, the flame retardant level of the sample reached V0. This flame retardant level can meet the needs of most use scenarios. Notably, when 15% flame retardant was added, the LOI was greatly improved compared to that of the blank control group, but the vertical combustion grade still did not reach the lowest flame retardant level. Thus, for use in a scenario where the flame retardant level requirement reaches V1, the addition of 20% flame retardant should be selected, not only to reduce the amount of flame retardant and the cost of materials but also to meet the material requirements.

### 3.2. Cone Calorimetry Test

**Figure 4:**
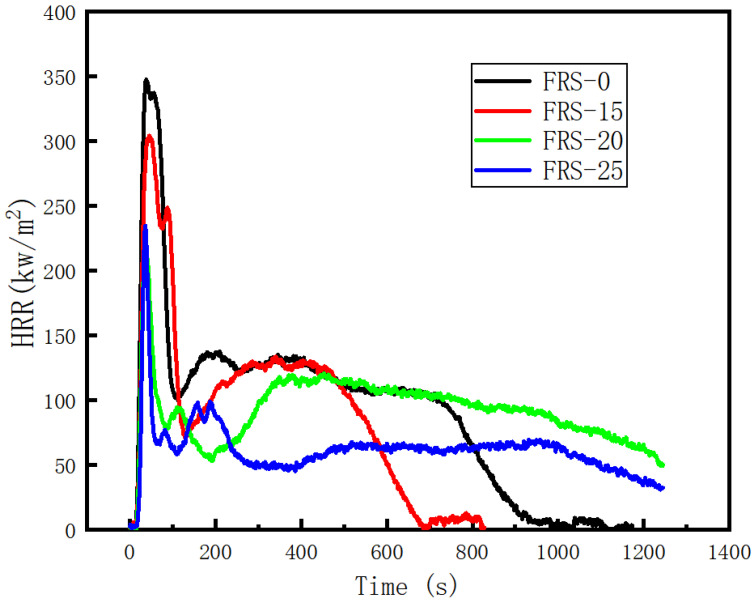
Comparison of combustion heat release rate of each group of flame-retardant corrugated plates.

The heat release rate of the corrugated plates with different amounts of added flame retardant is shown in Figure 4. For the corrugated plates without flame retardant addition, the heat of combustion quickly reached a peak; with increasing flame retardant addition, the maximum heat release rate decreased significantly compared with when no flame retardant was added. When 15%, 20%, and 25% flame retardant were each added, the maximum heat release rate decreased by 12.5%, 32.4%, and 37.4%, respectively. When the amount of flame retardant was increased from 15 to 25%, the effect on the reduction of the exothermic peak was significant. However, beyond 20% addition, the flame retardant had a limited effect on the reduction of the heat release peak. The total heat release was 121.8 when no flame retardant was added. When 15%, 20%, and 25% flame retardant were each added, the total heat release was 101.9, 93.1, and 80.3, corresponding to a decrease of 16.3%, 23.5%, and 34.1%, respectively. The total heat release reduction data demonstrate that the effect of increasing the flame retardant content on the total heat release and heat release peak was different. When the flame retardant content was increased from 20% to 25%, the total heat release was significantly reduced from 23.5% to 34.1%, which can also be reflected in the heat release rate diagram. For several groups of specimens, the heat release curve quickly peaks after ignition, after which the heat release rate decreases rapidly, leading to a second heat release peak.

The fire growth index (FGI) and the fire performance index (FPI) are commonly used to quantify and compare the size of the fire hazard. The FGI is the ratio of the heat release peak to the peak time; the larger the index, the faster the fire grows. The fire performance index is defined as the ratio of the ignition time to the heat release peak; the longer the ignition time, the smaller the heat release peak, and the larger the FPI value, reflecting the better flame retardant performance of the material. Many studies have shown that the FPI has a certain correlation with the development time of a fire in a closed space. The larger the FPI, the longer the boom. The boom time is an important parameter for designing fire escape methods. The FGI and the FPI of each group of corrugated plates are calculated and summarized in Table 4.

**Figure 5:**
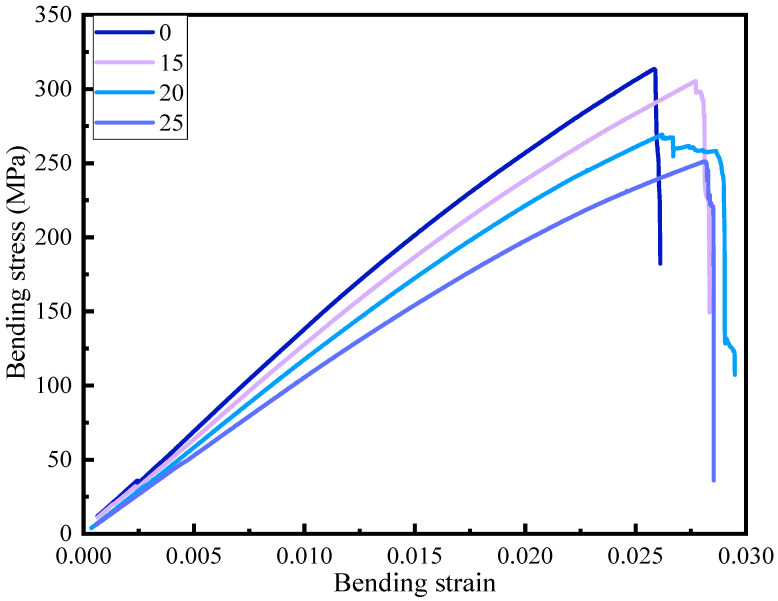
Bending stress-strain curves for each group of samples.

**Figure 6:**
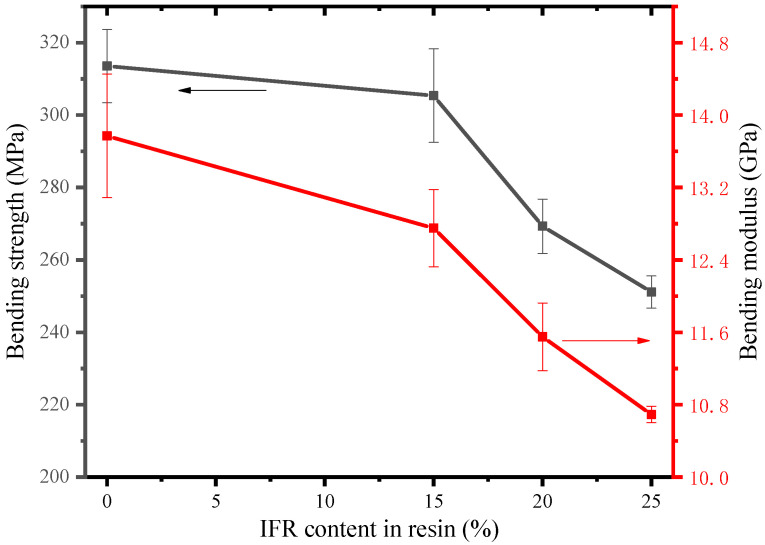
Comparison of flexural strength and flexural modulus of each group of samples.

The mechanical performance test is reflected through the bending strength test, and the bending test can better reflect the influence of flame retardants on the mechanical properties of flame-retardant prepreg. Figure 5 is the bending strength-strain curve, and Figure 6 is the line chart of the bending strength and bending modulus of various groups. Overall, the addition of flame retardants will result in a decrease in material bending strength, with the bending strength of the blank control group at 313.5 MPa and the bending modulus at 13.7 GPa. The bending strengths at 15%, 20%, and 25% flame retardant addition are 305.3, 269.2, and 251.1 MPa, respectively, representing a decrease of 2.6%, 14.1%, and 19.9% compared to the control group. The bending modulus at 15%, 20%, and 25% flame retardant addition are 12.7, 11.5, and 10.7 GPa, representing a decrease of 7.3%, 16.1%, and 21.9% compared to the control group’s modulus of 13.7 GPa. The addition of flame retardants will affect the mechanical properties of the material. When the flame retardant content is 15%, the decrease in the bending strength of the material is relatively small, but when the flame retardant content is further increased, the mechanical properties of the material decrease significantly. This is because when the amount of flame retardant added is too large, it will affect the adhesive strength between the matrix and the fibers at the micro level, resulting in a significant decrease in mechanical properties at the macro level.

From Figure 5, it can be seen that when the flame retardant content is 15%, the decrease in bending strength is not very significant, while the fracture toughness increases. When the flame retardant content is 20%, the bending strain reaches 0.025, and the bending strength of the material slightly decreases, then maintains the bending strength, and eventually, as the bending strain increases, the material fails and fractures. When the flame retardant content reaches 25%, the strain at which the material fails is comparable to that of 15% addition. By comparing the above sets of data, it can be concluded that the addition of flame retardants improves the material’s bending fracture toughness. The increase in toughness shows a trend of first increasing and then decreasing with the increase in the amount of flame retardants added.

## 4. Conclusions

1. The hot pressing scheme for preparing corrugated plates was explored. The skin and core material of the corrugated plate were prepared by the hot pressing method. The skin and core material were welded together by the hot melt method, which not only ensures the quality of the forming process but also has high production efficiency. The production and preparation process can be further optimized and improved in subsequent research.
2. The amount of flame retardant added to the corrugated plate can be determined according to the needs of different use scenarios to achieve a balance of the flame retardant performance, molding process, and cost-effectiveness of the material. The flame retardant efficiency is the highest with the addition of 20% flame retardant. This can meet the primary flame retardant demand while reducing the amount of flame retardant added and can significantly reduce the cost of materials, thereby ensuring the economic viability of the flame-retardant corrugated plates.
3. The use of flame retardant will have a certain impact on the mechanical properties of the material, but by optimizing the molding process and selecting the appropriate amount of flame retardant, the mechanical properties can meet the requirements of the corresponding grade when the flame retardant performance meets the requirements of the corresponding rates.
4. The flame retardant performance of the corrugated plate can be further designed because the skin and core material of the corrugated plate are formed separately. The amount of flame retardant added to the core material and the skin can be different to achieve a balance between flame retardant properties, mechanical properties, and the economic cost of materials.

## Figures and Tables

**Table 1:** Raw materials used in the experiment.

Raw Materials	Provider	Product Grade
Polypropylene	SK	Bx3900
Polypropylene	LyondellBasell	MF650X
MAPP	Exxon Mobil	Exxelor PO 1020
Continuous fiberglass yarn	Chongqing International Co.	4305s
IFR	Xinxiu Chemical	IFR-PP-1

**Table 2:** Specifications of devices used in the experiment.

Device	Manufacturer	Device Model
Prepreg belt production M	Designed and assembled in laboratory	BUAA-2019
Molding machine	DiDa Machinery Manufacturing Factory (Chengdu, China)	Y35-100T
Limiting oxygen index tester	Jiangning Analytical Instrument Co., Ltd. (Nanjing, China)	JF-3
Combustion grade tester	Yaoke (Shanghai, China)	YK-Y0142
Cone calorimeter	VOUCH (Shanghai, China)	6810

**Table 3:** Vertical combustion grade of each group of specimens.

Sample	UL-94 Rating	Dripping
CGF/PP	No rating	NO
CGF/PP/15IFR	No rating	NO
CGF/PP/20IFR	V1	NO
CGF/PP/25IFR	V0	NO

**Table 4:** Comparison of relevant data of various groups of flame-retardant corrugated plates.

Sample Name	FRS-0	FRS-15	FRS-20	FRS-25
Weight (g)	46.3	44.7	47.6	46.9
Heat release peak (kw/m^2^)	347.7	304.3	234.7	217.5
Total heat release (J)	121.8	101.9	93.1	80.3
Time to heat release peak (s)	29	34	24	26
Time to Ignition (s)	10	12	11	13
FPI	0.0287	0.0394	0.0469	0.0598
FGI	11.99	8.95	9.78	8.36

## Data Availability

Data are contained within the article.
